# Effect of extended close-up period duration on peripartum behavior, udder morphology, body condition score, and early lactation yield in primiparous Holstein cows

**DOI:** 10.14202/vetworld.2026.1642-1653

**Published:** 2026-04-25

**Authors:** Amel Najjar, Haroun Dougaz, Wael Halawa, Mousa Khalil, Naceur M’Hamdi

**Affiliations:** 1 Laboratory of Animal Genetic and Feed Resources LR15AGR01, National Agronomic Institute of Tunisia, University of Carthage, 1082 Tunis, Tunisia; 2 National Agricultural Research Center, Palestinian Ministry of Agriculture, Ramallah, West Bank, Palestine

**Keywords:** body condition score, close-up period, early lactation, Holstein cows, milk yield, peripartum behavior, primiparous dairy cows, udder morphology

## Abstract

**Background and Aim:**

The transition period is a critical phase in dairy cows, characterized by profound physiological, metabolic, and behavioral adaptations that influence health, welfare, and productivity. The duration of the close-up period, typically the final phase of the dry period before calving, plays a key role in preparing cows for parturition and subsequent lactation. However, the optimal length of this period remains debated, particularly in primiparous Holstein cows, where parity-specific responses may differ from those in multiparous animals. This study aimed to evaluate the effects of extending the close-up period beyond the conventional duration on peripartum behavior, calving characteristics, reproductive performance, body condition score (BCS), udder morphology, and early lactation yield.

**Materials and Methods:**

A prospective cohort study with retrospective grouping was conducted on 14 clinically healthy primiparous Holstein cows monitored from four weeks prepartum to four weeks postpartum. Based on actual close-up duration, cows were classified into Period A (18–26 days; n = 7) and Period B (27–35 days; n = 7). Behavioral and ordinal data were analyzed using the Mann–Whitney U test, while continuous variables such as gestation length and calving-to-first insemination interval were analyzed using a General Linear Model (GLM). Milk yield was assessed using both a GLM and a linear mixed-effects model with repeated-measures, with Bonferroni adjustment. Statistical significance was set at p < 0.05.

**Results:**

Behavioral parameters did not differ significantly between groups (p > 0.05). Period B cows exhibited a longer gestation length (279.0 ± 3.28 vs. 271.3 ± 4.17 days; p = 0.041) and a tendency toward a shorter calving-to-first insemination interval (p = 0.052). BCS was significantly higher in Period B at calving (p = 0.024) and at Week 4 postpartum (p = 0.004). Udder morphology improved in Period B, with higher udder position (p = 0.018) and vascularization scores both prepartum (p = 0.031) and postpartum (p = 0.012). Milk yield was markedly higher in Period B (36.80 ± 1.34 vs. 26.80 ± 0.96 L/day; p < 0.001), with consistent superiority across all postpartum weeks (p < 0.012).

**Conclusion:**

Extending the close-up period to 27–35 days in primiparous Holstein cows enhances physiological preparedness, improves udder morphology, and significantly increases early lactation milk yield without adversely affecting calving characteristics. These findings support the implementation of a moderately extended close-up period as a practical management strategy to optimize transition performance and productivity.

## INTRODUCTION

The peripartum period in dairy cows, encompassing the weeks before and after calving, is widely recognized as a critical phase that significantly influences animal health, productivity, and welfare [1, 2]. This transition period involves substantial physiological adaptations, including changes in metabolism, immune function, hormonal regulation, and behavior, which collectively affect calving outcomes and the success of subsequent lactation [3-6]. Traditionally, the dry period, the interval between the end of lactation and the next calving, is approximately 60 days, with the last 21 days designated as the close-up period to allow for mammary gland involution and metabolic adjustments [7, 8]. While the dry period length determines overall rest and recovery time, the close-up period specifically targets pre-calving metabolic and physiological preparation. Recent studies have challenged the universality of the traditional 21-day close-up period, indicating that altering its length can significantly affect calving ease, milk yield, and cow behavior [9–11]. Extending the close-up period beyond 21 days may provide additional time for metabolic stabilization and udder recovery, potentially reducing dystocia and improving early lactation performance [12]. However, prolonged close-up periods may also increase risks such as overconditioning or hormonal imbalance, negatively affecting pregnancy length, calving outcomes, and postpartum health [13, 14]. Behavioral changes during the peripartum period, including feeding patterns and social interactions, are further indicators of cow well-being that can be influenced by management strategies [15, 16].

Despite extensive research on transition period management in dairy cows, the optimal duration of the close-up period remains inadequately defined, particularly for primiparous Holstein cows. Most existing studies have focused on overall dry period length or have pooled primiparous and multiparous cows, thereby overlooking parity-specific physiological and metabolic differences that may influence transition outcomes. In addition, previous investigations have largely emphasized production parameters such as milk yield and metabolic health, whereas relatively little attention has been paid to integrated outcomes, including behavioral responses, udder morphology, and body condition dynamics during the peripartum period. Evidence regarding the impact of extending the close-up period beyond the conventional 21 days is inconsistent: some studies suggest improved metabolic adaptation and lactation performance, whereas others report potential risks, such as overconditioning and altered gestation characteristics. Furthermore, data generated under Mediterranean climatic conditions remain scarce, despite the known influence of environmental stressors on transition physiology and productivity. The lack of comprehensive, parity-specific, and environment-sensitive studies that simultaneously evaluate behavioral, physiological, reproductive, and lactational parameters highlights a critical knowledge gap. This gap limits the development of precise and evidence-based management strategies tailored to primiparous cows during the transition period.

This study was designed to address these limitations by evaluating the effect of extending the close-up period beyond the conventional duration on multiple dimensions of peripartum performance in primiparous Holstein cows. Specifically, the study aimed to compare two close-up periods (18–26 days and 27–35 days) and assess their effects on behavioral responses, calving characteristics, reproductive indices, body condition score (BCS), udder morphology, and early lactation milk yield. By integrating behavioral, physiological, and production-related outcomes within a single experimental framework, the study sought to provide a comprehensive understanding of how close-up period duration influences transition adaptation. In addition, by focusing exclusively on primiparous cows under Mediterranean farm conditions, the study aimed to generate parity-specific and context-relevant insights that can support more precise and practical transition management strategies. Ultimately, the findings are intended to contribute to optimizing animal welfare, improving lactation performance, and refining close-up period management in modern dairy production systems.

## MATERIALS AND METHODS

### Ethical approval

The experiment was conducted in accordance with Tunisian legal and institutional requirements for the care and use of animals in research. All procedures complied with the provisions of the Tunisian Livestock Law No. 2005-95 of 18 October 2005 and were performed by trained personnel in accordance with nationally accepted standards of good animal husbandry and welfare. Ethical review and approval for the study were obtained from the Institutional Animal Care and Use Committee under Certificate No. 108-CEEA-ENMV/25. The study was conducted under commercial farm conditions using clinically healthy primiparous Holstein cows, and all monitoring procedures, including behavioral observation, body condition scoring, udder assessment, reproductive follow-up, and milk yield recording, were noninvasive and conducted as part of routine herd management or with minimal animal disturbance. Transfer of cows to individual calving pens was performed only at the onset of parturition signs to ensure safe calving and close observation, and postpartum handling was performed in accordance with standard farm practice. No unnecessary stress, pain, or harmful experimental intervention was imposed on the animals during the study.

### Study location and period

The study was conducted on a commercial dairy farm in the Bizerte governorate of northern Tunisia (~40 km from Tunis) from February to June 2025, spanning late winter, spring, and early summer. The region has a sub-humid Mediterranean climate characterized by hot, dry summers and mild, wet winters, with an average annual temperature of 18.4°C and annual precipitation of 600–800 mm. During the study period, cows were managed under Mediterranean conditions with moderate seasonal heat stress typical of the spring-to-early-summer transition.

### Study design

This study was designed as a prospective cohort study with retrospective grouping to investigate the effect of close-up period duration on calving, behavior, and peripartum performance in primiparous Holstein dairy cows. The experimental timeline spanned from four weeks prepartum to four weeks postpartum. Primary outcomes included calving process, lactation performance, and reproductive parameters, while secondary outcomes included behavior, udder position, vascularization, and BCS during the peripartum period.

### Animals and experimental design

The experiment was conducted as an observational field study on fourteen (n = 14) clinically healthy primiparous Holstein dairy cows, monitored from four weeks before the expected calving date (W -4 to calving) through the four weeks of lactation (W 1 to W 4). During the close-up period, two intervals were defined based on the number of days preceding calving: Period A, ranging from 18 to 26 days pre-calving (n = 7), and Period B, ranging from 27 to 35 days pre-calving (n = 7). Allocation to each group was not randomized and was determined retrospectively based on the actual time of the cows’ entry into the close-up facility relative to their subsequent calving dates. At entry into the close-up period, all cows in both groups exhibited a BCS between 2.75 and 3.25 on a 5-point scale.

This approach was chosen because under farm conditions, strict randomization of close-up periods was not feasible. The prospective cohort with retrospective grouping allowed us to study naturally occurring differences in close-up duration while keeping all other management factors identical.

### Feeding

Dry cows were offered a basal diet composed of 4 kg of grass silage, 3 kg of hay, and 3 kg of concentrate per day. During the close-up period, this ration was supplemented daily with 10 g of rumen-protected methionine and 100 g of β-carotene. In the final stage of the close-up period, each cow additionally received a daily oral dose of 300 mL monopropylene glycol. Supplementation with rumen-protected methionine, β-carotene, and propylene glycol was intended to support metabolic adaptation, liver function, and energy balance during the close-up period, thereby promoting a smooth transition to lactation. The basal diet and supplements were formulated to meet the nutrient requirements of dry and close-up primiparous Holstein cows according to INRAE [17] recommendations, providing adequate energy, protein, and fiber to support metabolic adaptation during the transition period. As the precise calving date was determined retrospectively, supplementation commenced five days before the expected calving date, estimated from artificial insemination (AI) records. Despite minor discrepancies between predicted and actual calving dates, the supplementation protocol was uniformly applied to ensure consistency and standardization of treatment across all animals.

Feed intake was assumed to be equal between the two groups, as all cows received the same diet and supplementation and were housed under identical conditions. All supplements were discontinued at calving, and cows were subsequently managed according to the standard lactation diet of the herd.

### Housing

Pregnant cows were housed in a barn comprising common straw-bedded areas and individual calving pens, which were used upon the appearance of the first signs of parturition. Cows were moved into these pens to calve in a calm and hygienic environment. Bedding in the calving pens was renewed daily. Housing conditions provided adequate space allowance and appropriate stocking density, consistent with standard commercial dairy management practices. The barn and calving pens were predominantly illuminated by natural daylight. Artificial lighting (low-intensity LED aisle lights) was used only for routine night checks; all behavioral and conformation assessments were performed in natural daylight.

Following calving, lactating cows were transferred to the main barn. The barn was a free-stall type, featuring a central feed alley, two circulation alleys, and two large resting areas with straw bedding. Each section was equipped with an automatic concentrate dispenser and two water troughs providing continuous access to drinking water. Calving pens allowed visual and limited physical contact with conspecifics, ensuring social interaction while facilitating individual monitoring during the peripartum period. Daily barn maintenance included mechanical scraping of the circulation alleys to remove manure and bedding renewal using a straw blower.

### Calving process

Upon the appearance of the first signs of imminent parturition, the cow was transferred to a clean, straw-bedded individual calving pen located near the dry cow area. The calving process was evaluated using a three-point scale (0 to 2) as described by Roche et al. [18]: A score of 0 indicated an easy calving, with no complications or assistance required; A score of 1 corresponded to a moderately difficult calving, with minor difficulties requiring minimal assistance; A score of 2 denoted a difficult or dystocic calving, involving major complications and requiring human intervention to ensure the safety of both the dam and the calf. All calving observations and any required interventions were conducted according to a standardized protocol and were consistently applied across all animals. Behavioral observations were recorded from the onset of the pre-calving phase, particularly the onset of restlessness, the duration of fetal expulsion, and the time to placental expulsion. After 48 hours, the cow was moved to the main barn for postpartum monitoring.

### Behavioral assessment

Behavioral assessment was conducted during the peripartum period. Behavioral indicators were selected based on their relevance to peripartum welfare. Indicators of human–animal relationship were recorded based on the animal’s response to human approach, including eye contact, flight response, or acceptance. Eye expression was also analyzed as a reflection of the animal’s emotional state. Additional indicators of general behavior were observed, including the frequency of ear and tail movements and bellowing. Behavioral observations were conducted once weekly using a 10-minute focal sampling session per cow in the morning prior to feed distribution, following the Welfare Quality® protocol [19]. The observation period was considered sufficient to capture key behavioral patterns while minimizing disturbance to the animals. Observations were conducted by the same trained observer throughout the study to ensure consistency and to minimize observational bias.

### Reproductive parameters

The dates of the previous calving and the preceding fertile insemination were obtained from farm records for cows in both groups. Actual gestation length was calculated based on the interval between the date of the preceding fertile insemination and the actual calving date. Fertile insemination was initially confirmed by non-return to estrus and subsequently verified by transrectal ultrasonography performed at approximately 30 days post-insemination.

Postpartum resumption of ovarian activity was assessed by transrectal palpation, together with estrus detection and the calving-to-first-insemination interval, defined as the number of days between the actual calving date and the first insemination attempt following calving. Estrus detection was based on visual observation of behavioral signs, including standing estrus, increased activity, and mounting behavior. AI using frozen–thawed semen was performed approximately 12 h after estrus detection.

### Udder position and vascularization

The evaluation of udder position and vascularization was conducted once a week via direct visual and tactile inspection of each animal while in a standing, relaxed posture. Evaluations were conducted by the same trained examiner, under natural daylight conditions, before milking and according to the standard guidelines of the International Committee for Animal Recording [20, 21], which were applied without modification. The examiner followed a standardized scoring protocol, ensuring consistency and reliability of udder assessments throughout the study. The anatomical position of the udder was scored according to established morphological criteria, focusing on its relative placement relative to the hock joints and the abdominal wall. A semi-quantitative scale (ranging from 0 to 9) was applied to classify udder joints: A score of 0 corresponded to a very pendulous udder, markedly below hock level and at risk of contacting the ground; a score of 1 indicated an extremely low udder, significantly hanging below the hocks; a score of 2 described a low udder, clearly below hock level but without extreme laxity; a score of 3 referred to a slightly low udder, marginally below the hocks; a score of 4 indicated an udder positioned approximately at hock level; a score of 5 was assigned when the udder was slightly elevated above the hocks; a score of 6 described a moderately high udder, visibly suspended above hock level and showing a balanced conformation; a score of 7 indicated a high udder, well attached and closely aligned to the abdominal wall; a score of 8 represented a very high udder, compact and positioned close to the abdomen. Finally, a score of 9 referred to an extremely high udder, tightly suspended and nearly flush with the abdominal surface. Vascularization was assessed by inspection and manual palpation of the superficial epigastric veins. A semi-quantitative scale, ranging from 0 to 3, was applied as follows: A score of 0 indicated the absence of visible or palpable venous structures; a score of 1 reflected slight vascular development, with thin, straight veins barely visible or perceptible to touch, and a slow refill after compression; a score of 2 corresponded to moderate vascularization, with veins clearly visible or palpable, showing moderate tortuosity and refilling within a few seconds; a score of 3 indicated a high degree of vascular prominence, with large, tortuous, highly visible veins that refilled rapidly after compression, suggestive of strong mammary blood flow.

### Lactation

Milking was performed twice daily in a milking parlor (Delaval, Tumba, Sweden) at 4:00 AM and 2:00 PM. Milk yield was measured during both morning and evening milking sessions using automated milk meters. Meters were calibrated routinely according to the manufacturer’s schedule. Raw per-milking data were automatically captured by the parlor software; no manual transcription was used for the analyses. Unless otherwise stated, the postpartum analytical window for weekly models comprised Weeks 1–4 postpartum, as shown in Table 5.

### BCS

BCS was visually assessed every week throughout the peripartum period. Scoring was performed using a 1-to-5 scale as described by Wildman et al. [22], where a score of 1 indicates an emaciated condition and a score of 5 corresponds to an excessively fat condition. BCS was assessed by a single trained scorer throughout the study to maximize repeatability. Scoring was conducted without knowledge of the eventual retrospective group classification (close-up groups were assigned after calving based on the actual days spent in the preparation area). To minimize postural and feeding effects, BCS evaluations were performed during morning rounds, outside of milking and not immediately after feed delivery.

### Statistical analysis

All statistical analyses were performed using IBM SPSS Statistics for Windows, Version 25.0 (IBM Corp., Armonk, NY, USA) to evaluate the effect of the duration of the pre-calving preparation period on behavioral, physiological, reproductive, and lactation parameters of dairy cows. Cows were classified into two groups according to the length of the preparation period: Period A (18–26 days) and Period B (27–35 days).

Behavioral variables and ordinal scores, including human–animal interaction, eye expression, ear movement, tail movement, and bellowing frequency, were summarized as medians (Interquartile Range, IQR). Differences between groups for these non-normally distributed variables were assessed using the Mann–Whitney U test.

BCS, udder position, and udder vascularization scores at different time points relative to calving were also expressed as median (IQR) and compared between groups using the Mann–Whitney U test. When applicable, pairwise differences within the same time point were indicated using different superscript letters.

Gestation length and the interval from calving-to-first AI were analyzed as continuous variables and are presented as mean ± standard error (SE). These parameters were compared between groups using a General Linear Model (GLM), with preparation period included as a fixed effect.

Calving difficulty was treated as a categorical variable and expressed as percentages for each score category. Group differences in the distribution of calving difficulty were analyzed using Fisher’s exact test. Durations of calving-related stages, including the preparatory phase, fetal expulsion, placental expulsion, and total calving duration, were summarized as medians (IQRs) and compared between groups using the Mann–Whitney U test due to non-normal distributions and limited sample size.

Lactation performance was assessed using two complementary approaches. Mean total milk yield per cow, averaged across the postpartum weeks, was analyzed using a GLM with preparation period as a fixed effect, and results are presented as mean ± SE. Morning and evening milk yields were summarized descriptively without inferential testing.

To evaluate weekly changes in milk yield and account for repeated measurements within cows, a linear mixed model (LMM) was applied. The model included Group, Week, and their interaction (Group × Week) as fixed effects, with cow included as a random effect. A first-order autoregressive covariance structure [AR(1)] was used to model within-cow correlations across weeks. Estimated marginal means (EM, Means ± SE) were obtained, and pairwise comparisons between groups within each week were adjusted using the Bonferroni correction. Significant differences were denoted by different superscript letters.

All statistical tests were two-sided, and statistical significance was declared at p < 0.05.

## RESULTS

### General behavior

The duration of the close-up period did not significantly affect most general behavioral parameters of dairy cows (Table 1). Human–animal interaction scores were identical between Period A and Period B, with median values of 0.0 (IQR: 0.0 – 1.0) in both groups (p = 0.911). Similarly, no significant differences were observed between groups for ear movement frequency, tail movement frequency, or bellowing frequency (p > 0.05). Eye expression scores did not differ significantly between the two preparation periods, with both groups showing a median score of 2.0; however, the IQR was slightly wider in Period A (1.0 – 3.0) than in Period B (p = 0.908). Overall, behavioral indicators suggested that extending the close-up period had no measurable impact on general behavioral responses.

**Table 1:** Variation in the general behavior of dairy cows according to the duration of pre-calving preparation.

Parameter	Period A	Period B	p-value
Human–animal interaction	0.0 (0.0-1.0)	0.0 (0.0-1.0)	0.911
Eye expression (Score)	2.0 (1.0-3.0)	2.0 (1.0-3.0)	0.908
Ear movement (Frequency)	7.0 (5.0-8.0)	7.0 (5.0-8.0)	0.406
Tail movement (Frequency)	5.0 (3.0-7.0)	5.0 (3.0-7.0)	0.295
Bellowing (Frequency)	2.0 (1.0-3.0)	2.0 (1.0-3.0)	0.181

A: Close-up period [18 – 26 days]; B: close-up period [27 – 35 days] Data are presented as median (IQR).

### BCS and udder morphology

BCS varied significantly between groups at specific time points (Table 2). No significant difference was detected between Period A and Period B during the prepartum phase (Week −3; p = 0.875). However, at calving (Week 0), cows in Period B exhibited a significantly higher BCS compared with Period A (median: 3.50 vs. 3.25; p = 0.024). This difference persisted into the postpartum period (Week 4), where Period B cows maintained higher BCS values than Period A cows (median: 3.25 vs. 2.75; p = 0.004).

**Table 2:** Variation in body condition score and udder morphology.

Parameter / Time point	Period A	Period B	p-value
Body condition score			
Week -3 (Pre-partum)	3.50 (3.2–3.7)	3.55 (3.3–3.8)	0.875
Week 0 (Calving)	3.25 (3.0–3.5)^b^	3.50 (3.3–3.6)^a^	0.024
Week 4 (Postpartum)	2.75 (2.5–3.0)^b^	3.25 (3.0–3.5)^a^	0.004
Udder Position (Score)			
Week -1 (Pre-partum)	3.0 (2.0–4.0)	3.0 (3.0–4.0)	0.520
Week 2 (Postpartum)	2.0 (1.5–2.5)^b^	3.0 (2.5–3.5)^a^	0.018
Udder Vascularization			
Week -1 (Pre-partum)	2.0 (1.0–2.5)^b^	3.0 (2.0–3.0)^a^	0.031
Week 2 (Postpartum)	2.0 (2.0–3.0)^b^	3.0 (3.0–4.0)^a^	0.012

A: preparation period [18–26 days]; B: preparation period [27–35 days] median (IQR); a,b within a row differ significantly (p < 0.05).

Udder morphology parameters were also influenced by the close-up period. Udder position scores did not differ between groups during the prepartum assessment (Week −1; p = 0.520), but a significant difference emerged postpartum (Week 2), with higher udder position scores observed in Period B compared with Period A (p = 0.018). Likewise, udder vascularization scores were significantly greater in Period B both prepartum (Week −1; p = 0.031) and postpartum (Week 2; p = 0.012), indicating enhanced vascular development associated with the longer close-up period.

### Calving characteristics and reproductive parameters

Gestation length differed significantly between the two groups (Table 3). Cows in Period B exhibited a longer gestation length compared with those in Period A (279.0 ± 3.28 vs. 271.3 ± 4.17 days; p = 0.041).

**Table 3:** Variation in calving difficulty and durations.

Parameter	Period A	Period B	p-value
Gestation length (day)	271.3 ± 4.17^b^	279 ± 3.28^a^	0.041
Calving difficulty			
Score 0 (%)	75	70	0.783
Score 1 (%)	12.5	20	
Score 2 (%)	12.5	10	
Duration of the preparatory phase (min)	80 (60-95)	75 (60-105)	0.937
Duration of fetal expulsion (min)	55 (40-80)	50 (30-70)	0.562
Duration of placental expulsion (min)	215 (180-250)	240 (200-330)	0.933
Total duration of calving (min)	350 (310-390)	390 (315-450)	0.591
Calving-to-first AI (day)	57.60 ± 15.80	50.90 ± 14.70	0.052

A: close-up period [18–26 days]; B: close-up period [27–35 days] Data are presented as percentages for categorical variables (calving difficulty scores), median (IQR) for non-normally distributed continuous variables (calving duration parameters), and mean ± standard error for gestation length and calving-to-first artificial insemination interval. Categorical variables were analyzed using Fisher’s exact test. Continuous variables related to calving duration were analyzed using the Mann–Whitney U test due to small sample size and non-normal distribution. Gestation length and calving-to-first AI interval were analyzed using a General Linear Model. All statistical tests were two-sided, and statistical significance was set at p < 0.05.

The distribution of calving difficulty scores did not differ significantly between groups (p = 0.783). Most cows in both groups experienced unassisted calving (Score 0), accounting for 75% in Period A and 70% in Period B. No significant differences were detected in the duration of the preparatory phase, fetal expulsion, placental expulsion, or total calving duration between the two preparation periods (p > 0.05 for all comparisons).

The interval from calving-to-first AI tended to be shorter in Period B compared with Period A; however, this difference did not reach statistical significance (p = 0.052).

### Lactation performance

Mean total milk yield per cow, averaged across postpartum weeks, was significantly higher in cows subjected to the longer close-up period (Table 4). Period B cows produced substantially more milk compared with Period A cows (36.80 ± 1.34 vs. 26.80 ± 0.96 L; p < 0.001).

**Table 4:** Variation in lactation parameters of dairy cows during the postpartum period.

Parameter	Period A	Period B	p-value
Mean total milk yield (L) per cow (average across weeks)	26.80 ± 0.96 ^b^	36.80 ± 1.34 ^a^	<0.001
Morning milk yield (L)	13.60 ± 0.35	20.50 ± 0.49	Descriptive
Evening milk yield (L)	10.90 ± 0.34	16.30 ± 1.0	Descriptive

A: close-up period [18–26 days]; B: close-up period [27–35 days]. Values are presented as mean ± standard error of the mean. The comparison of mean total milk yield per cow was performed using a General Linear Model with preparation period as a fixed effect. All other parameters are presented as descriptive statistics only. Statistical significance was declared at p < 0.05.

Morning and evening milk yields were descriptively higher in Period B than in Period A; however, these parameters were not subjected to inferential statistical testing and are therefore reported for descriptive purposes only.

### Weekly milk yield dynamics

Weekly milk yield analysis using an LMM revealed consistent and significant differences between groups across all evaluated weeks (Table 5). In each postpartum week, cows in Period B demonstrated higher EM of daily milk yield compared with cows in Period A (p = 0.012 after Bonferroni adjustment).

**Table 5:** LMM-based weekly estimated marginal means of milk yield with Bonferroni-adjusted pairwise comparisons between groups.

Week	Period A	Period B	Pairwise P (Bonferroni)
1	26.10 ± 1.10 ^b^	31.00 ± 1.20 ^a^	0.012
2	25.80 ± 1.17 ^b^	34.50 ± 1.33 ^a^	<0.001
3	26.90 ± 1.52 ^b^	36.29 ± 1.40 ^a^	<0.001
4	27.53 ± 1.19 ^b^	38.26 ± 1.62 ^a^	<0.001

A: close-up period [18–26 days]; B: close-up period [27–35 days]. Values are Estimated marginal mean ± standard error of the mean. LMM included Group, Week, and Group × Week as fixed effects, with cow as a random effect and AR(1) covariance. Bonferroni-adjusted pairwise comparisons were applied within week. Different letters denote significant differences at p < 0.05. LMM = linear mixed model.

Specifically, the difference between groups was evident from Week 1 and persisted through Week 4, indicating a stable and sustained advantage in milk production associated with the longer close-up period. The presence of significant group differences across all weeks supports a robust effect of preparation period on lactation performance over time.

## DISCUSSION

This study assessed whether extending the close-up period from 18–26 days (Period A) to 27–35 days (Period B) influences peripartum behavior, BCS, and udder morphology, calving characteristics, reproductive indices, and early lactation performance in primiparous Holstein cows. Under uniform housing and feeding management, the longer close-up duration was primarily associated with improved physiological status (BCS), more favorable udder scores, and markedly higher milk yield, while general behavioral indicators and calving process variables remained broadly similar between groups. These outcomes support the view that transition period management can shape metabolic and mammary preparedness for lactation [1-3].

### General behavior

In the present dataset, general behavioral parameters did not differ between close-up durations (Table 1). Human–animal interaction scores, eye expression, ear and tail movements, and bellowing frequency were comparable in Period A and Period B. The absence of detectable behavioral differences suggests that, within the tested range, close-up duration alone may be insufficient to shift the behavioral indicators captured by the Welfare Quality®-based focal observations. Behavior around calving is multifactorial and can be strongly influenced by facility characteristics, social context, and environmental conditions, which may override modest changes in prepartum duration [16, 23–25]. Moreover, several studies indicate that peripartum behavioral alterations (e.g., rumination, activity, feeding changes) are particularly informative when monitored continuously or with high temporal resolution, especially in relation to metabolic strain and postpartum disorders [5, 26–28]. The weekly focal sampling approach used here, while standardized and minimally invasive, may have limited sensitivity to detecting transient behavioral changes occurring in the immediate pre-calving window [29, 30]. Collectively, the behavioral findings imply that both close-up strategies provided broadly comparable welfare conditions as reflected by the selected indicators.

### BCS and udder morphology

A clear physiological distinction between groups emerged in BCS (Table 2). Although BCS did not differ at Week −3, cows in Period B had higher BCS at calving (Week 0) and maintained higher BCS at Week 4 postpartum, indicating better preservation of body reserves across the transition. This pattern is consistent with the central role of the transition period in determining energy balance at the onset of lactation; improved prepartum adaptation can help mitigate early lactation negative energy balance and support performance [1–4, 31]. Importantly, the BCS values remained within a moderate range, suggesting improved condition without clear evidence of excessive overconditioning in this cohort, though this remains an important management consideration given links between higher BCS and metabolic risk [2, 3, 13].

Udder traits were also improved by the longer close-up duration. Udder position was significantly higher in Period B at Week 2 postpartum, and udder vascularization scores were significantly higher in Period B both prepartum (Week −1) and postpartum (Week 2) (Table 2). These findings suggest that a longer close-up period may support udder conformation and superficial mammary vascular development during the peripartum window. Biologically, mammary tissue remodeling and preparation for lactation are highly active during the dry and close-up phases; management that optimizes this interval can influence udder function and subsequent milk output [7, 8]. The observed vascularization differences are also coherent with evidence linking mammary blood flow and udder tissue characteristics to lactational performance [32]. While the present study relied on standardized field scoring [21], the concordant improvements in udder position and vascularization support a plausible pathway whereby extended close-up duration enhances mammary readiness.

### Calving characteristics and reproductive parameters

Cows in Period B exhibited a significantly longer gestation length than those in Period A (Table 3). Gestation length variability can reflect complex interactions among genetics, parity, maternal status, and environmental or management factors, and it may shift with changes in transition physiology [2, 4, 6]. However, in the present study, this difference did not translate into detectable disadvantages in the calving process.

Additionally, the durations of the preparatory phase, fetal expulsion, placental expulsion, and total calving did not differ (Table 3). This indicates that extending the close-up duration within the tested range did not increase the risk of dystocia or prolong parturition. Such a result is consistent with literature emphasizing that parturition difficulty is often driven more by fetal–maternal size relationships, pelvic anatomy, and parity-related factors than by moderate adjustments in transition management [10, 14, 33].

For postpartum reproductive recovery, the calving-to-first AI interval tended to be shorter in Period B (p = 0.052; Table 3). Although not statistically significant, the direction aligns with the concept that improved metabolic adaptation during the transition can support earlier resumption of ovarian function and better reproductive efficiency [2–4, 13]. Given the limited sample size, this result should be interpreted as suggestive rather than confirmatory.

### Lactation performance

The most pronounced and consistent effect of close-up duration was observed in milk yield. Period B cows had a substantially higher mean total milk yield across postpartum weeks than Period A (Table 4). This finding is consistent with evidence that transition period and dry period strategies influence early lactation output through effects on mammary remodeling, endocrine adaptation, and energy balance [7–9, 11]. Under conditions of adequate nutrition, longer or conventional dry period management has been associated with better production outcomes than shortened strategies [35–38]. In the present study, the higher milk yield in Period B is also consistent with the parallel observations of higher BCS at calving and more favorable udder scores, which together suggest improved metabolic and mammary preparedness at the onset of lactation [1, 4, 32].

### Weekly milk yield dynamics

The mixed model analysis confirmed that the milk yield advantage in Period B was not transient: Period B exceeded Period A in every postpartum week assessed, with significant Bonferroni-adjusted pairwise differences throughout (Table 5). This sustained divergence supports a robust effect of close-up duration on the early lactation trajectory. Such persistence is biologically plausible because management-driven differences in prepartum adaptation can influence the magnitude of negative energy balance, metabolic stability, and mammary secretory capacity over several weeks postpartum [3, 4, 9]. The consistency across weeks strengthens the inference that the longer close-up period improved lactational readiness rather than producing an isolated short-term response.

### Overall interpretation, implications, and limitations

Overall interpretation and implications as a whole, and consistent with the results sequence, extending the close-up period to 27–35 days in primiparous Holstein cows produced a coherent pattern of advantages concentrated in physiological readiness and lactational output: while general behavioral indicators remained comparable between groups (suggesting broadly similar welfare expression under the selected observational approach), cow in Period B showed higher BCS at calving and during early postpartum, together with more favorable udder position and vascularization scores, indicating improved transition adaptation and mammary preparedness [1–4, 8, 21, 32]. Although gestation length was longer in Period B, this shift was not accompanied by increased calving difficulty or prolonged calving stages, supporting the interpretation that the longer close-up window did not compromise parturition outcomes within the tested range, in line with the multifactorial determinants of calving ease described in the literature [10, 14, 33]. The most robust and practically relevant effect was the marked and sustained improvement in milk yield, confirmed both by higher overall mean production and by consistently higher weekly EM across the early postpartum weeks, which accords with evidence that close-up management can influence early lactation performance through effects on metabolic stability and mammary remodeling [7, 9, 11, 35, 37]. Accordingly, under the nutritional and housing conditions applied, a moderately extended close-up period appears to be a feasible management option to enhance early lactation efficiency without detriment to calving outcomes, while recognizing that the observational design and limited sample size warrant confirmation in larger cohorts with integrated metabolic and reproductive biomarkers [13, 29–31].

This study has some limitations that should be acknowledged. Methodological limitations include the small sample size (n = 14 primiparous cows) and the retrospective grouping based on actual close-up days, which may allow for residual confounding. Metabolic and health biomarkers (e.g., NEFA, BHBA, calcium) and milk composition/SCC were not measured. Results should therefore be interpreted cautiously and considered hypothesis-generating, warranting confirmation in larger, prospectively balanced cohorts with integrated metabolic and milk quality endpoints. Future studies including a greater number of animals and multiple herds are recommended to validate these results.

## CONCLUSION

The present study demonstrates that extending the close-up period from 18–26 days to 27–35 days results in measurable improvements in peripartum performance in primiparous Holstein cows. Cows in Period B maintained higher BCS at calving and during early postpartum, exhibited improved udder position and vascularization scores, and produced substantially greater milk yield throughout the first 4 weeks of lactation. Weekly LMM analysis confirmed a consistent production advantage in Period B across all postpartum weeks. In contrast, general behavioral indicators, calving difficulty distribution, and durations of calving stages remained comparable between groups, indicating that the extended close-up period did not adversely affect calving dynamics. Although gestation length was increased in Period B, the calving-to-first AI interval showed a tendency toward improvement, suggesting a potential positive effect on reproductive recovery.

From a practical perspective, these findings support implementing a moderately extended close-up period (27–35 days) as a feasible and effective transition management strategy to enhance early lactation performance without compromising calving outcomes. The approach is particularly relevant for primiparous cows, where improved BCS stability and udder development may contribute to better metabolic adaptation and sustained milk production. The consistency of results under uniform feeding and housing conditions further reinforces the applicability of this strategy in commercial dairy systems.

The main strength of this study lies in its integrated evaluation of behavioral, physiological, reproductive, and lactational parameters within a single experimental framework, combined with repeated-measures analysis of milk yield, which provides robust insight into early lactation dynamics. However, several limitations should be acknowledged. The small sample size (n = 14) and retrospective grouping design may limit generalizability and introduce potential confounding. In addition, the absence of metabolic biomarkers (e.g., NEFA, BHBA, Ca) and milk composition data restricts deeper interpretation of the physiological mechanisms underlying the observed effects.

Future studies should include larger, prospectively balanced cohorts across multiple herds to validate these findings and enhance external applicability. Incorporation of metabolic, endocrine, and milk quality indicators, along with continuous behavioral monitoring, would provide a more comprehensive understanding of transition physiology. Comparative studies involving multiparous cows are also warranted to determine whether the observed benefits are parity-specific or applicable at the herd level.

In conclusion, extending the close-up period to 27–35 days is a promising management strategy to improve BCS, udder morphology, and early lactation milk yield in primiparous Holstein cows without negatively affecting calving performance, thereby improving transition efficiency and dairy productivity.

## DATA AVAILABILITY

The raw data supporting the findings of this study are available on request from the corresponding author.

## AUTHORS’ CONTRIBUTIONS

AN: Conceptualization, study design, methodology, data collection, statistical analysis, and writing – original draft. HD: Data collection, data management, and statistical analysis. WH: Methodology refinement and validation of results. MK: Conceptual input, statistical analysis, data interpretation, data curation, and critical review. NM: Methodology, data curation, critical review, validation of results, and final editing. All authors have read and approved the final manuscript and agreed to be accountable for all aspects of the work.
